# Correction: Pronominal anaphora resolution in Polish: Investigating online sentence interpretation using eye-tracking

**DOI:** 10.1371/journal.pone.0296557

**Published:** 2024-01-02

**Authors:** Agata Wolna, Joanna Durlik, Zofia Wodniecka

There are errors in the Funding section. The correct Funding statement is: This research was possible thanks to the grant SONATA BIS grant no 2015/18/E/HS6/00428 awarded to ZW by the National Science Center Poland (https://ncn.gov.pl/). During writing the paper JD was supported by Etiuda scholarship no 2018/28/T/HS6/00277 awarded by the Polish National Science Centre (https://ncn.gov.pl/). The open access license of the publication was funded by the Priority Research Area Society of the Future under the programme “Excellence Initiative–Research University” at the Jagiellonian University in Krakow. The funders had no role in study design, data collection and analysis, decision to publish, or preparation of the manuscript.
