# Supplementary material for: Pronominal anaphora resolution in Polish: Investigating online sentence interpretation using eye-tracking
Source: PLoS One. 2022 Jan 11;17(1):e0262459. doi: 10.1371/journal.pone.0262459 (PMC8752000; doi:10.1371/journal.pone.0262459)
Supplement: S1 Appendix — (DOCX) [file pone.0262459.s001.docx]

**Appendix A**

**Experimental stimuli for Experiment 2**

*Nu/S* – null pronoun, subject antecedent

*Nu/O* – null pronoun, object antecedent

*Ov/S –* overt pronoun, subject antecedent

*Ov/O* – overt pronoun, object antecedent

1. *Nu/S* Artystka pracowała z dziewczynkami, kiedy przyjechała na warsztaty plastyczne.

*Nu/O* Artystki pracowały z dziewczynką, kiedy przyjechała na warsztaty plastyczne.

*Ov/S* Artystka pracowała z dziewczynkami, kiedy ona przyjechała na warsztaty plastyczne. *Ov/O* Artystki pracowały z dziewczynką, kiedy ona przyjechała na warsztaty plastyczne.

*The artist/artists worked with a girl/girls when Ø/she came to the art workshop.*

1. *Nu/S* Mężczyzna zadzwonił do kolegów, kiedy trafił na oddział ratunkowy.

*Nu/O* Mężczyźni zadzwonili do kolegi, kiedy trafił na oddział ratunkowy.

*Ov/S* Mężczyzna zadzwonił do kolegów, kiedy on trafił na oddział ratunkowy.

*Ov/O* Mężczyźni zadzwonili do kolegi, kiedy on trafił na oddział ratunkowy.

*The man/men called a friend/friends when Ø/he came into the E.R.*

1. *Nu/S* Pielęgniarz plotkował z lekarzami, kiedy wychodził ze szpitala wieczorem.

*Nu/O* Pielęgniarze plotkowali z lekarzem, kiedy wychodził ze szpitala wieczorem.

*Ov/S* Pielęgniarz plotkował z lekarzami, kiedy on wychodził ze szpitala wieczorem.

*Ov/O* Pielęgniarze plotkowali z lekarzem, kiedy on wychodził ze szpitala wieczorem.

*The nurse/nurses gossiped with a doctor/doctors when Ø/he was leaving the hospital in the evening.*

1. *Nu/S* Dziennikarka rozmawiała z aktorkami, kiedy wyszła z premiery spektaklu.

*Nu/O* Dziennikarki rozmawiały z aktorką, kiedy wyszła z premiery spektaklu.

*Ov/S* Dziennikarka rozmawiała z aktorkami, kiedy ona wyszła z premiery spektaklu.

*Ov/O* Dziennikarki rozmawiały z aktorką, kiedy ona wyszła z premiery spektaklu

*A journalist/Journalists talked to the actress/actresses when Ø/she came out of the premiere.*

1. *Nu/S* Piłkarze spoglądali na kolegę, kiedy opowiadał o strzelonej bramce.

*Nu/O* Piłkarz spoglądał na kolegów, kiedy opowiadał o strzelonej bramce.

*Ov/S* Piłkarz spoglądał na kolegów, kiedy on opowiadał o strzelonej bramce.

*Ov/O* Piłkarze spoglądali na kolegę, kiedy on opowiadał o strzelonej bramce.

*The player/players looked at the friend/friends when Ø/he was talking about the goal he scored.*

1. *Nu/S* Staruszka zadzwoniła do sąsiadek, kiedy wychodziła do sklepu po zakupy.

*Nu/O* Staruszki zadzwoniły do sąsiadki, kiedy wychodziła do sklepu po zakupy.

*Ov/S* Staruszka zadzwoniła do sąsiadek, kiedy ona wychodziła do sklepu po zakupy.

*Ov/O* Staruszki zadzwoniły do sąsiadki, kiedy ona wychodziła do sklepu po zakupy.

*The old woman/women called the neighbour/neighbours when Ø/she was going out to the store.*

1. *Nu/S* Tłumaczka napisała do przełożonych, kiedy dostała prestiżową nagrodę.

*Nu/O* Tłumaczki napisały do przełożonej, kiedy dostała prestiżową nagrodę.

*Ov/S* Tłumaczka napisała do przełożonych, kiedy ona dostała prestiżową nagrodę.

*Ov/O* Tłumaczki napisały do przełożonej, kiedy ona dostała prestiżową nagrodę.

*A translator/Translators wrote to the supervisor/supervisors when Ø/she was awarded a prestigious prize.*

1. *Nu/S* Gospodarz pomachał do gości, kiedy zajrzał do dużego salonu.

*Nu/O* Gospodarz pomachał do gości, kiedy on zajrzał do dużego salonu.

*Ov/S* Gospodarze pomachali do gościa, kiedy zajrzał do dużego salonu.

*Ov/O* Gospodarze pomachali do gościa, kiedy on zajrzał do dużego salonu.

*The host/hosts waived to the guest/guests when Ø/he looked into a big living room.*

1. *Nu/S* Maturzysta rozmawiał z nauczycielami, kiedy wyszedł z egzaminu z polskiego.

*Nu/O* Maturzyści rozmawiali z nauczycielem, kiedy wyszedł z egzaminu z polskiego.

*Ov/S* Maturzysta rozmawiał z nauczycielami, kiedy on wyszedł z egzaminu z polskiego.

*Ov/O* Maturzyści rozmawiali z nauczycielem, kiedy on wyszedł z egzaminu z polskiego.

*A high school graduate/High school graduates was/were talking to the teacher/teachers when Ø/he came out of the Polish exam.*

1. *Nu/S* Pielęgniarka dyskutowała z pacjentkami, kiedy wchodziła do gabinetu zabiegowego.

*Nu/O* Pielęgniarki dyskutowały z pacjentką, kiedy wchodziła do gabinetu zabiegowego.

*Ov/S* Pielęgniarka dyskutowała z pacjentkami, kiedy ona wchodziła do gabinetu zabiegowego.

*Ov/O* Pielęgniarki dyskutowały z pacjentką, kiedy ona wchodziła do gabinetu zabiegowego.

*The nurse/nurses were arguing with a patient/patients when Ø/she was coming out of the treatment room.*

1. *Nu/S* Księgowa dzwoniła do szefowych, kiedy pojechała na szkolenie informatyczne.

*Nu/O* Księgowe dzwoniły do szefowej, kiedy pojechała na szkolenie informatyczne.

*Ov/S* Księgowa dzwoniła do szefowych, kiedy ona pojechała na szkolenie informatyczne.

*Ov/O* Księgowe dzwoniły do szefowej, kiedy ona pojechała na szkolenie informatyczne.

*The accountant/accountants called the boss/bosses when Ø/she went to an IT training.*

1. *Nu/S* Trener rozmawiał z siatkarzami, kiedy decydował o nowych strojach.

*Nu/O* Trenerzy rozmawiali z siatkarzem, kiedy decydował o nowych strojach.

*Ov/S* Trener rozmawiał z siatkarzami, kiedy on decydował o nowych strojach.

*Ov/O* Trenerzy rozmawiali z siatkarzem, kiedy on decydował o nowych strojach.

*The coach/coaches talked to the volleyball player/players when Ø/he was deciding on new outfits.*

1. *Nu/S* Artysta rozmawiał z gośćmi, kiedy przyszedł na wernisaż wystawy.

*Nu/O* Artyści rozmawiali z gościem, kiedy przyszedł na wernisaż wystawy.

*Ov/S* Artysta rozmawiał z gośćmi, kiedy on przyszedł na wernisaż wystawy.

*Ov/O* Artyści rozmawiali z gościem, kiedy on przyszedł na wernisaż wystawy.

*The artist/artists talked with the guest/guests when Ø/he came to the opening of the exhibition.*

1. *Nu/S* Dłużnik negocjował z wierzycielami, kiedy przyszedł na rozprawę sądową.

*Nu/O* Dłużnicy negocjowali z wierzycielem, kiedy przyszedł na rozprawę sądową.

*Ov/S* Dłużnik negocjował z wierzycielami, kiedy on przyszedł na rozprawę sądową.

*Ov/O* Dłużnicy negocjowali z wierzycielem, kiedy on przyszedł na rozprawę sądową.

*The debtor/debtor was/were negotiating with the creditor/creditors when Ø/he came to the court hearing.*

1. *Nu/S* Sprzątaczka narzekała na szefowe, kiedy wyszła na popołudniową przerwę.

*Nu/O* Sprzątaczki narzekały na szefową, kiedy wyszła na popołudniową przerwę.

*Ov/S* Sprzątaczka narzekała na szefowe, kiedy ona wyszła na popołudniową przerwę.

*Ov/O* Sprzątaczki narzekały na szefową, kiedy ona wyszła na popołudniową przerwę.

*The cleaner/cleaners was/were complaining about the boss/bosses when Ø/she left for an afternoon break.*

1. *Nu/S* Sprzedawczyni spojrzała na kobiety, kiedy otwierała drzwi do sklepu.

*Nu/O* Sprzedawczynie spojrzały na kobietę, kiedy otwierała drzwi do sklepu.

*Ov/S* Sprzedawczyni spojrzała na kobiety, kiedy ona otwierała drzwi do sklepu.

*Ov/O* Sprzedawczynie spojrzały na kobietę, kiedy ona otwierała drzwi do sklepu.

*The saleswoman/saleswomen looked at the woman/women when Ø/she unlocked the door to the store.*

1. *Nu/S* Projektant współpracował z dostawcami, kiedy prowadził w centrum firmę.

*Nu/O* Projektanci współpracowali z dostawcą, kiedy prowadził w centrum firmę.

*Ov/S* Projektant współpracował z dostawcami, kiedy on prowadził w centrum firmę.

*Ov/O* Projektanci współpracowali z dostawcą, kiedy on prowadził w centrum firmę.

*The designer/designers collaborated with the supplier/suppliers when Ø/he was running a business in the center.*

1. *Nu/S* Ogrodnik dyskutował z szefami, kiedy zauważył na pomidorach szkodniki.

*Nu/O* Ogrodnicy dyskutowali z szefem, kiedy zauważył na pomidorach szkodniki.

*Ov/S* Ogrodnik dyskutował z szefami, kiedy on zauważył na pomidorach szkodniki.

*Ov/O* Ogrodnicy dyskutowali z szefem, kiedy on zauważył na pomidorach szkodniki.

*The gardener/gardeners was/were arguing with the boss/bosses when Ø/he noticed pests on the tomatoes.*

1. *Nu/S* Przedszkolanka podeszła do rodziców, kiedy przyszła do przedszkola rano.

*Nu/O* Przedszkolanki podeszły do rodzica, kiedy przyszedł do przedszkola rano.

*Ov/S* Przedszkolanka podeszła do rodziców, kiedy ona przyszła do przedszkola rano.

*Ov/O* Przedszkolanki podeszły do rodzica, kiedy on przyszedł do przedszkola rano.

*The kindergarten teacher/teachers approached the parent/parents when Ø/(s)he came to kindergarten in the morning.*

1. *Nu/S* Prezenterka spojrzała na autorki, kiedy usiadła w fotelu w studio.

*Nu/O* Prezenterki spojrzały na autorkę, kiedy usiadła w fotelu w studio.

*Ov/S* Prezenterka spojrzała na autorki, kiedy ona usiadła w fotelu w studio.

*Ov/O* Prezenterki spojrzały na autorkę, kiedy ona usiadła w fotelu w studio.

*The presenter/presenters looked at the author/authors when Ø/she sat down in an armchair in the studio.*

1. *Nu/S* Skrzypek spojrzał na dyrygentów, kiedy położył na pulpicie nuty.

*Nu/O* Skrzypkowie spojrzeli na dyrygenta, kiedy położył na pulpicie nuty.

*Ov/S* Skrzypek spojrzał na dyrygentów, kiedy on położył na pulpicie nuty.

*Ov/O* Skrzypkowie spojrzeli na dyrygenta, kiedy on położył na pulpicie nuty.

*The violinist/violinists looked at the conductor/conductors as Ø/he laid notes on the lectern.*

1. *Nu/S* Dziewczyna dzwoniła do koleżanek, kiedy pojechała na zakupy do galerii.

*Nu/O* Dziewczyny dzwoniły do koleżanki, kiedy pojechała na zakupy do galerii.

*Ov/S* Dziewczyna dzwoniła do koleżanek, kiedy ona pojechała na zakupy do galerii.

*Ov/O* Dziewczyny dzwoniły do koleżanki, kiedy ona pojechała na zakupy do galerii.

*The girl/girls called a friend/friends when Ø/she went shopping at the gallery.*

1. *Nu/S* Student podszedł do wykładowców, kiedy wychodził z zajęć z logiki.

*Nu/O* Studenci podeszli do wykładowcy, kiedy wychodził z zajęć z logiki.

*Ov/S* Student podszedł do wykładowców, kiedy on wychodził z zajęć z logiki.

*Ov/O* Studenci podeszli do wykładowcy, kiedy on wychodził z zajęć z logiki.

*The student/students approached the lecturer/lecturers when Ø/he was leaving a logic class.*

1. *Nu/S* Nastolatek wpadł na znajomych, kiedy wyprowadzał na spacer psa.

*Nu/O* Nastolatki wpadły na znajomego, kiedy wyprowadzał na spacer psa.

*Ov/S* Nastolatek wpadł na znajomych, kiedy on wyprowadzał na spacer psa.

*Ov/O* Nastolatki wpadły na znajomego, kiedy on wyprowadzał na spacer psa.

*A teenager/Teenagers ran into a friend/friends when Ø/he was walking a dog.*

1. *Nu/S* Poseł zadzwonił do współpracowników, kiedy pracował nad nową ustawą.

*Nu/O* Posłowie zadzwonili do współpracownika, kiedy pracował nad nową ustawą.

*Ov/S* Poseł zadzwonił do współpracowników, kiedy on pracował nad nową ustawą.

*Ov/O* Posłowie zadzwonili do współpracownika, kiedy on pracował nad nową ustawą.

*The MP/MPs called a colleague/colleagues when Ø/he was working on the new law.*

1. *Nu/S* Dziewczynka pisała do ciotek, kiedy mieszkała w innym mieście.

*Nu/O* Dziewczynki pisały do ciotki, kiedy mieszkała w innym mieście.

*Ov/S* Dziewczynka pisała do ciotek, kiedy ona mieszkała w innym mieście.

*Ov/O* Dziewczynki pisały do ciotki, kiedy ona mieszkała w innym mieście.

*The girl/girls wrote to the aunt/aunts when Ø/she was living in another city.*

1. *Nu/S* Wykładowca spojrzał na studentów, kiedy wchodził do sali wykładowej.

*Nu/O* Wykładowcy spojrzeli na studenta, kiedy wchodził do sali wykładowej.

*Ov/S* Wykładowca spojrzał na studentów, kiedy on wchodził do sali wykładowej.

*Ov/O* Wykładowcy spojrzeli na studenta, kiedy on wchodził do sali wykładowej.

*The lecturer/lecturers looked at the student/students when Ø/he entered the lecture hall.*

1. *Nu/S* Trenerka patrzyła na nastolatki, kiedy pływała w głębokim basenie.

*Nu/O* Trenerki patrzyła na nastolatkę, kiedy pływała w głębokim basenie.

*Ov/S* Trenerka patrzyła na nastolatki, kiedy ona pływała w głębokim basenie.

*Ov/O* Trenerki patrzyły na nastolatkę, kiedy ona pływała w głębokim basenie.

*The trainer/trainers watched the teen/teens when Ø/she swam in the deep pool.*

1. *Nu/S* Policjant pomachał do chłopców, kiedy przechodził przez ruchliwe skrzyżowanie.

*Nu/O* Policjanci pomachali do chłopca, kiedy przechodził przez ruchliwe skrzyżowanie.

*Ov/S* Policjant pomachał do chłopców, kiedy on przechodził przez ruchliwe skrzyżowanie.

*Ov/O* Policjanci pomachali do chłopca, kiedy on przechodził przez ruchliwe skrzyżowanie.

*The policeman/policemen waved at the boy/boys when Ø/he was crossing a busy intersection.*

1. *Nu/S* Ciocia stała obok siostrzenic, kiedy kupowała na straganie kwiaty.

*Nu/O* Ciocie stały obok siostrzenicy, kiedy kupowała na straganie kwiaty.

*Ov/S* Ciocia stała obok siostrzenic, kiedy ona kupowała na straganie kwiaty.

*Ov/O* Ciocie stały obok siostrzenicy, kiedy ona kupowała na straganie kwiaty.

*The aunt/aunts was/were standing next to the niece/nieces when Ø/she was buying flowers from a market stall.*

1. *Nu/S* Architekt rozmawiał z inżynierami, kiedy przyjechał na inspekcję budowy.

*Nu/O* Architekci rozmawiali z inżynierem, kiedy przyjechał na inspekcję budowy.

*Ov/S* Architekt rozmawiał z inżynierami, kiedy on przyjechał na inspekcję budowy.

*Ov/O* Architekci rozmawiali z inżynierem, kiedy on przyjechał na inspekcję budowy.

*The architect/architects was/were talking to the engineer/engineers when Ø/he arrived for the construction inspection.*

1. *Nu/S* Przewodnik wpadł na turystów, kiedy dotarł do hotelu po obiedzie.

*Ov/S* Przewodnik wpadł na turystów, kiedy on dotarł do hotelu po obiedzie.

*Nu/O* Przewodnicy wpadli na turystę, kiedy dotarł do hotelu po obiedzie.

*Ov/O* Przewodnicy wpadli na turystę, kiedy on dotarł do hotelu po obiedzie.

*The guide/guides ran into a tourist/tourists when Ø/he arrived at the hotel after lunch.*
