# Supplementary material for: Pronominal anaphora resolution in Polish: Investigating online sentence interpretation using eye-tracking
Source: PLoS One. 2022 Jan 11;17(1):e0262459. doi: 10.1371/journal.pone.0262459 (PMC8752000; doi:10.1371/journal.pone.0262459)
Supplement: Attachment — Submitted filename: Wolna et al_review.pdf [file pone.0262459.s002.pdf]

Pronominal anaphora resolution in Polish: investigating online sentence interpretation using eye-tracking.

The present study replicates a previous study on the pronoun resolution preferences in Spanish (Chamorro et al., 2015). The authors of the present study set out to investigate the interpretation strategies for null and overt subject pronouns in another null-subject language, namely Polish. In order to do so, they make use of two experimental tasks, an offline sentence-interpretation task, where participants had to decide on the referent of an ambiguous pronoun and subsequently rate the naturalness of the sentence, and an online eye-tracking during reading task where participants read sentences, which they also had to rate for their naturalness afterwards. The main findings of the study are the following:

- Offline experiment:
  Polish speakers prefer to interpret the null pronoun as co-referential with the subject antecedent, and the overt pronoun as co-referential with the object antecedent.

- Online experiment:
  Finding #1: the interpretation of a null pronoun is not disrupted by a violation of its natural preference (for the subject antecedent); however, the interpretation of the overt pronoun is disrupted when it is forced to co-refer with the subject antecedent.

  Finding #2: the sentences where the (null/overt) pronoun refers to the antecedent in the object position incur an additional processing cost as compared with the sentences where the pronoun refers to the subject antecedent.

In my opinion, the present study presents several strong points: First, it is a close replication of a previous study (both in terms of tasks and materials); second, it adds a (Slavic) language to the repertoire of languages that have been studied in the pronoun resolution literature; third, the reported results confirm a well-known bias previously attested in other languages.

My concern regarding this article, as I explain just below, has to do with the fact that the literature review and parts of the discussion section are rather superficial (in particular when it comes to the discussion of the factors that play a role in pronoun resolution).

My recommendation is that the article is accepted after some revisions have been made.

These revisions have mainly to do with the discussion of the linguistic factors that play a role in pronoun resolution in the literature review and in the discussion/conclusion sections. The authors discuss in certain detail the issue of antecedent prominence as being crucial in pronoun resolution and, in relation to this, they discuss the syntactic function/position of the subject as being an important contributor of this prominence (this is reinforced by the discussion of Carminati's Position of Antecedent Hypothesis and the related psycholinguistic findings on languages such as Italian and Spanish). However, the discussion stops there. No mention of other potential crucial factors (e.g. topicality, thematic role, order of mention, etc.) is to be found. The authors do evoke multiple times discourse-pragmatic factors (see the non-exhaustive list below of the sections where this comes up) but they never give examples of any these factors nor do they discuss any previous studies that investigated them.

The discussion of these additional factors is paramount in order to be able to better account for the present findings. This seems to be the case regarding the finding that the sentences where a (null/overt) pronoun refers to the antecedent in the object position incur an additional processing cost compared with the sentences where the pronoun refers to the subject antecedent, which the authors account in the following terms: "This suggests that Polish speakers interpret sentences under the working assumption that they refer to the most prominent subject antecedent" (p. 27, line 640-645). What we see in this case (i.e. in the object conditions) is a shift in the sentence topic from the main clause to the subordinate clause. This dispreference for a topic-shift intra-sententially has also been attested in other languages, such as French, German, and Spanish (see Colonna et al., 2012, 2015; de la Fuente, 2016; Patterson & Felser, 2020). The notion of (sentence) topic and these previous findings need to be discussed in the paper.

My second (more general) remark concerns the choice of manipulation regarding the disambiguating part of the experimental sentences in the online experiment, namely the agreement between the subject of the main clause and the verb of the subordinate clause (a manipulation also used by Chamorro et al. 2015 in their experiment in Spanish). The pronoun resolution strategies commonly observed in the literature (null pronoun refers to a subject antecedent, overt pronoun refers to an object antecedent) remain *preferences* which can be easily overridden by other factors (this could indeed explain why there is certain variability in the literature regarding the resolution preference for Spanish null and overt pronouns, for example). Morpho-syntactic cues such as gender and number agreement between pronoun and antecedent and subject-verb agreement are examples of such strong cues which can override the role of other factors in pronoun resolution (like the position of the antecedent). That is why the differences between conditions 1 and 2, on the one hand, and conditions 3 and 4, on the other hand, are not always robust.

In relation to this, I was not very surprised to read that the interpretation of a null pronoun is not disrupted by a violation of its natural preference (for the subject antecedent); however, the interpretation of the overt pronoun is disrupted when it is forced to co-refer with the subject antecedent (contrary to what was previously found in the literature). For me, there is a difference in terms of acceptability between the two "less-natural" conditions 1 and 4: Condition 1 (overt pronoun/subject match) seems a lot less natural than condition 4 (null pronoun/object match). This has to do, on the one hand, with what I described in the paragraph above regarding subject-verb agreement and, on the other hand, with the fact that the overt pronoun is a lot more (pragmatically) marked and not always pragmatically felicitous in short decontextualized sentences.

<u>Mention of syntax vs. pragmatic/discourse factors:</u>
(p. 2) "Overall, in the Polish language, interpretation of a null pronoun seems to be more sensitive to pragmatic cues of reference than syntactic cues of reference, while resolution of overt pronouns relies strongly on syntax-based cues."
(p. 3) "their reference relies not only on the rigid rules of the syntax of a given language but is also sensitive to pragmatic and contextual factors"
(p. 3) "the mechanisms of anaphora resolution can be sensitive to syntactic and also pragmatic or contextual factors"

(p. 4) (discourse-oriented language) "other sources of information such as discourse and context become much more important for the correct resolution of an anaphoric expression"
(p. 6) "differences between languages that are driven by language-specific, syntax-based, pragmatic constraints"
(p. 10, line 216) "stem from divergent sensitivity of null and overt pronouns to syntactic and discourse-related or context-related determinants of the prominence of an antecedent"
(p. 11, line 251) "can be prone to the influence of other non-syntactic factors. These factors…"
(p. 16, line 387-388) "we should be able to assess the sensitivity of null and overt pronouns to the syntax-based determinants of the prominence of an antecedent"
(p. 17, line 398-399) "can provide information on the sensitivity of pronouns to different types of prominence cues"
(p. 29, line 708-710) "relies on both syntactic and contextual factors"[…] "can be modulated by pragmatic and contextual cues"

(p. 8-9) "Whenever a violation of a preferred pronoun-antecedent match incurs additional processing cost, this indicates that the speaker is using a syntactic cue (i.e. the antecedent's syntactic position within a sentence) to identify the referent of an anaphorical pronoun. On the other hand, if violating the speaker's preference does not lead to additional processing cost, a speaker likely uses non-syntactic cues to define the prominence of an antecedent (i.e. context-related or discourse-related information) to interpret the anaphorical pronoun."

- ➔ What context-related or discourse-related information? It seems that "discourse" and "contextual" are used as synonymous terms in certain sections of the paper, and as two different things in other (e.g. on page 28, the use of the term "context-dependent information" seems to refer to the information on subject-verb agreement).
- ➔ Are there previous findings that show this clear-cut tendency? Is this related to the findings for Italian and Spanish by Filiaci and colleagues? Because there is cross-linguistic variation there and even variation within the same language (Spanish) if we take into account the results by Chamorro and colleagues.
- ➔ (p. 10, line 213) "the available empirical evidence demonstrates that the preference for anaphora resolution seems to be more universal for null pronouns" > taken into account the two languages under discussion in the literature review, this is not so clear cut, right? It seems to be the case for Italian, for not so much for Spanish.

<u>Other comments</u>

(p. 4) sentence-oriented languages (e.g. Italian, Polish, Spanish) rely on morpho-syntactic cues (subject-verb agreement) to establish the referent of an anaphorical expression. > Nothing about sentence vs. discourse oriented languages in the discussion (does Polish behave like a hybrid (sentence-oriented for null pronouns) but discourse-oriented for overt pronouns)?

(p. 3) The assertion that the pronoun *she* and the noun *Mary* in *When Mary crossed the street, she looked back at the monument* "denominate one and the same person" is misleading. Strictly speaking, *she* can also refer to somebody else other than *Mary* here.

(p. 4, 14, 20) Given the rich morphology of the language, the examples in Polish should also include a gloss in English.

(p. 5, line 90) “on the other hand, less-reduced expression, like those containing an overt pronoun” > “on the other hand, less-reduced expression, like an overt pronoun”

(p. 14, line 338-340) Did the filler items also feature null and overt pronouns? If so, I’m not sure I’m very comfortable with the fact that the filler items also included the critical (null vs. overt pronoun) manipulation.

If this was the case, I was also wondering whether the filler items also contained the same proportions of null and overt pronouns. This is an important point, as it was been previously shown that the varying proportions of the type of pronoun present in the experiment has a direct impact on the participants’ resolution preference (cf. Fernandes et al. 2018).

I’m also little concerned about the use of the different conjunctions for the filler sentences. We know from previous literature (the works by Kehler and Rhode, de la Fuente et al. 2016), that the kinds of coherence relations that these types of conjunctions establish between main and subordinate clause, have an impact on pronoun resolution. I wonder to what extent the filler items also influenced the resolution preference of the experimental items here.

<u>My point</u>: See my comment above about pronoun preference being easily overridden by other factors. Controlling for all these potential extraneous factors is, therefore, crucial.

(p. 15, line 344) “was modulated by the use of a pronoun” > “was modulated by the nature of the pronoun”

(p. 15, line 364-365) “which indicates that native Polish speakers find null pronoun sentences more natural than overt pronoun sentences” > This sounds a little too general. A better formulation would be “which indicates that native Polish speakers find the null pronoun condition more natural than the overt pronoun conditions” or “which indicates that native Polish speakers find the sentences containing a null pronoun more natural than those containing an overt pronoun”

(p. 17, line 406-412) Why would this be your prediction? As you said previously, the preference of the null pronoun for a subject antecedent seems to be more robust according to previous findings. The findings of Chamorro et al. are rather exceptional in that respect. Shouldn’t you then expect the opposite (i.e. that null pronouns are more sensitive to violations of the syntax-based bias)?

(p. 20, line 476-480) It is not clear to me whether there were 8 experimental items (in the 4 experimental conditions = 32 experimental sentences in total = 4 lists each with 8 experimental items and 64 filler items) or 32 experimental items (in the four experimental conditions = 128 experimental sentences in total = 4 lists each with 32 experimental items and 64 filler items).

(p. 21, line 509) Strictly speaking, the *go-past* measure is more of a "hybrid" measure that reflects both early *and* late processes.

(p. 26, summary and general discussion sections) The authors use multiple times formulations such as "processing sentences which do not refer to the most prominent antecedent" or "sentences referring to an antecedent in the object position always incur…" This sounds a little weird. Strictly speaking, it is the pronoun that refers to one or another antecedent not the sentence. A better formulation can be "sentences in which the (null/overt) pronoun refers to…".
