# Supplementary material for: Pronominal anaphora resolution in Polish: Investigating online sentence interpretation using eye-tracking
Source: PLoS One. 2022 Jan 11;17(1):e0262459. doi: 10.1371/journal.pone.0262459 (PMC8752000; doi:10.1371/journal.pone.0262459)
Supplement: Attachment — Submitted filename: Response to reviewers.docx [file pone.0262459.s004.docx]

We would like to thank the Editor and the Reviewers for their detailed comments and suggestions. We hope that the revisions in the manuscript and our accompanying responses successfully address the concerns of the Editor and the Reviewers and that after revision you will find our work suitable for publication in PLOS One.

Whenever in our responses we refer to specific pages and lines corresponding to the changes that we have introduced to the manuscript, the page and lines numbers refer to the marked-up version of the manuscript.

Yours sincerely,

Agata Wolna, Joanna Durlik, Zofia Wodniecka

**Response to the Editor’s comments:**

Please reword any unclear, misleading, or stylistically convoluted passages in your manuscript. Examples of these can already be found in your abstract:

"cues of reference" - odd wording

"an ambiguous sentence interpretation task" - this phrase is itself ambiguous

"First, in an ambiguous sentence interpretation task, we explored the natural biases that occur during the interpretation of null or overt pronouns." - What is meant by "natural" here (are there also unnatural biases)? Also, if this was an offline task, how can it possibly explore biases *during* interpretation?

"antecedents which are in the syntactic position of a subject or an object" - Why not simply say "subject or object antecedents"?

Please carefully check the remainder of your manuscript for further such instances and amend these. Reviewer #1 makes several helpful suggestions in this regard.

**Response:**

We have reviewed the manuscript and fixed all the unclear, misleading and (statistically) convoluted passages that we could identify. In the case of the examples that you mentioned in your comment:

- We reworded the formulation “cues of reference” wherever it appeared in this form to “factors determining the reference/prominence”; however the term “cues” (e.g. “discourse prominence cues”) is commonly used in the pronominal anaphora resolution literature so we decided to keep the word “cues” did not correct it in other instances
- We fixed the ambiguous phrase (“an ambiguous sentence interpretation task”) in the abstract. In the current version of the text it is as follows:

*“we explored the interpretation preferences of null and overt pronouns in an ambiguous sentence interpretation task”*

- We have replaced the more precise terms “antecedents in the syntactic position of subject/object” with the shorter version that you propose: “subject/object antecedent”. In a couple of instances we did not change the wording which stresses the syntactic position of an antecedent, but only if it was justified by the narrative(e.g. on p. 40 l. 953-954, where we believe that it is crucial to stress that the subjecthood/objecthood refers to a syntactic feature of an antecedent, because we build an argument that the unexpected processing cost observed in the post-critical AOI is actually related to the syntactic processing)
- We have incorporated in the text all other stylistic suggestions of Reviewer #1

**Response to the Reviewers’ comments**

**Reviewer #1**

I was reviewer 1 on a previous draft of this manuscript. My previous major concerns have been addressed satisfactorily, and I do not have any large objections, though I do have some comments on this revised version and some additional minor suggestions.

Comments:

- p. 9, l. 191-197: “For example, in the case of overt pronoun interpretation in Italian and Spanish, the increased processing cost resulting from forcing a shift of reference from the syntactic subject of a sentence (which is also the first-mentioned referent) to the syntactic object is observed for Italian but not for Spanish. Within the Form-Specific Multiple-Constraint Approach, this cross-linguistic difference can be explained by referring to the different sensitivity of pronouns to syntactico-semantic cues in both these languages: in Italian, pronoun resolution seems to rely more heavily on this type of cue than in Spanish.” But why are Italian overt pronouns more sensitive to syntactico-semantic cues than Spanish overt pronouns? “differences in pronouns’ reference resolution do not necessarily need to be associated with typological differences between languages” (l.186-187): this seems to be the case instead if the cross-linguistic difference in overt pronouns interpretation depends of the different sensitivity of overt pronouns in Italian and in Spanish. “differences between sentence-oriented and discourse-oriented languages can be accounted for by referring to the much higher sensitivity of pronouns to the informational structure of discourse in discourse-oriented languages than in sentence-oriented languages.”(l. 198-200): Here again, typological differences between languages are seen to be responsible for cross-linguistics differences in pronoun interpretation.

**Response 1.1:** Thank you very much for spotting this problem. In the fragment that the Reviewer found unclear, what we wanted to argue was that sensitivity to the syntactico-semantic cues may account for differences in pronoun resolution preference even between languages that are not typologically distant (i.e.: Spanish vs. Italian, in contrast to e.g. Spanish vs. Chinese). However, we agree with the Reviewer that different sensitivity to some types of cues can actually be considered to be a typological difference between the languages and as such our argument was somewhat circular.

As the considerations of typological distance between Spanish and Italian are not the central notion for the argument we want to make, from the beginning of the paragraph, we removed the sentence which claimed that **“**differences in pronouns’ reference resolution do not necessarily need to be associated with typological differences between languages”. In the current form, the paragraph (p.9 l. 189-206) does not differentiate between typological differences between the languages and the differences in sensitivity to syntactico-semantic cues. We believe that it solves the problem of circularity of the argument.

- Thematic roles are defined as the semantic roles (agent, patient,…) (see p. 6, l. 109-111). However, further on in the text, I do not understand the formulations: “the role of the thematic topic” (p. 40, l. 945) “and the thematic role of a topic” (p.41, l. 967). I would change to “the role of topic”. Furthermore, the authors propose to distinguish the syntacto-semantic cues from discourse-related cues but in the conclusion, they seem to include topicality in the syntacto-semantic cues (p. 41, l. 977) when I understood that they were talking about the discourse topic throughout the paper. This should be clarified.

**Response 1.2:** Thank you for spotting these inconsistencies.

We have corrected the formulations related to the role of topic on p. 42 l. 1000 and on p. 42 l. 1010. In both instances, “the thematic topic” / “thematic role of a topic” have been replaced with “the role of topic”, following your suggestion.

We have also reworded the conclusions so that they no longer claim that topicality is an instance of a syntactico-semantic cue (which was a mistake on our part). In the current formulation the fragment in question was changed to the following form:

*“Our results indicate that in Polish, syntactico-semantic cues - such as subjecthood and information structure (i.e., order of mention and thematic roles) - are the dominant constraints of the prominence of an antecedent”*

- In the discussion (p. 37-38), the authors discuss the role of the context of a sentence but again, the context was not manipulated in the current study. So, contrary to what the authors claim, I do not think that the online results allow them “to determine whether these preferences can be modulated by the context of a given sentence” (p. 37, l. 858). Just as I do not think that they can conclude that null pronouns “easily shift their reference towards a more contextually appropriate referent” (p. 38, l. 888) since null pronouns, whether disambiguated in favour of the object or the subject, were presented in the same contexts.

**Response 1.3:** Thank you for bringing up this unfortunate use of the term “context” . We agree with the Reviewer that as context was not manipulated in our study we cannot draw any conclusions related to how it influences the pronoun resolution. Whenever we used the term “context” we actually referred to the grammatical constraints of a sentence, which were manipulated in Study 2. We replaced all the mentions of “context” in the discussion with “grammatical constraints”.

The problematic formulations were changed in the text to the following:

*“[...] to determine whether these preferences can be modulated by* ***the grammatical constraints*** *of a given sentence”*

*“[...] null pronouns are forced to refer to an antecedent which does not follow the preferred antecedent match, they can easily shift their reference towards* ***a less-preferred, but grammatically correct referent****”*

- In further discussion (p. 41-42), the argument about the anti-focus effect needs clarification. To the best of my knowledge, contrary to what the authors claim (p. 40, l. 952-953), an increased processing cost associated with an anti-focus effect was not observed. The anti-focus effect is the fact that the pronoun refers preferentially to the topic of the current discourse unit (which is not the focused by cleft antecedent of the discourse unit in which it appears since the clefting function is to signal a topic shift for the subsequent discourse unit). The authors should clarify what they want to argue here.

**Response 1.4**: Thank you for spotting this inconsistency. It has been corrected: the section that mentioned it is supposed to refer, in a broader sense, to experiments that explored the focus effect in pronoun resolution. We do not aim to explain the anti-focus effect as it is not central to our argumentation so we have removed the mention of it. The corrected version of the paragraph is the following:

*“Previous studies on focus effect in anaphora resolution reported a similar effect: an increased processing cost associated with a topic shift towards a less-prominent antecedent which has been cued by the information structure of the current discourse (10,11,14,15). In this context, our results can be interpreted as showing that Polish speakers automatically interpret the most-prominent antecedent (the first available antecedent or the subject antecedent) to be the topic of a sentence, but as new information cues another antecedent which is coherent with the current discourse, the reference is shifted.”*

**Smaller suggestions:**

- p. 2, l. 19: in the abstract, change “we explored the natural biases that occur during the interpretation of null or overt pronouns” to “we explored the interpretation preferences of null and overt pronouns”

**Response 1.5:** This fragment has been changed according to the Reviewer’s suggestion.

- p. 9, l. 196, change “pronoun resolution seem” to “pronoun resolution seems”

**Response 1.6:** Thank you for spotting this mistake. It has been corrected in the text.

- p. 10, l. 235, change “the natural pronoun-antecedent match” to “the preferred pronoun-antecedent match”

**Response 1.7:** We changed this formulation according to the Reviewer’s suggestion.

- p. 11, l. 247, change “the natural anaphora resolution preference” to “the preferred anaphora interpretation”

**Response 1.8:** We changed this formulation according to the Reviewer’s suggestion.

- p. 11, l. 255, why is “followed” in italics?

**Response 1.9:** The format has been changed to a regular text.

- p. 11, l. 256, change “a violation of the natural preference” to “a violation of the offline preference”

**Response 1.10:** We changed this formulation according to the Reviewer’s suggestion.

- p. 13, l. 294, change “neither the preference” to “neither the preferences”

**Response 1.11:** Thank you for spotting this issue. It has been corrected in the text.

- p. 14, 332, in the title, change “What is the natural pattern of anaphora resolution in Native Polish speakers?” to “What is the offline pattern of anaphora resolution in Native Polish speakers?”

**Response 1.12:** We changed this formulation according to the Reviewer’s suggestion.

- p. 16, l. 366-367, change “In order to test whether a pronoun can influence the natural tendency to interpret the antecedent of a sentence” to “In order to test whether the pronoun form (null vs overt) can influence its interpretation preference”

**Response 1.13:** This fragment has been changed according to the Reviewer’s suggestion.

- p. 20, l. 477, change “violation of the natural preference for anaphora resolution” to “violation of the preferred anaphora interpretation”

**Response 1.14:** We changed this formulation according to the Reviewer’s suggestion.

- p. 27, l. 635, change “on native Polish speakers’ natural preference” to on native Polish speakers’ offline preference”

**Response 1.15:** We changed this formulation according to the Reviewer’s suggestion.

- p. 33, l. 758, change “the natural pattern of pronominal anaphora resolution in Polish” to “the pattern of pronominal anaphora resolution in Polish”

**Response 1.16:** We changed this formulation according to the Reviewer’s suggestion.

- p. 33, l. 759, change “we established a preference” to “we established the offline preference"

**Response 1.17:** We changed this formulation according to the Reviewer’s suggestion.

- p. 33, l. 768, change “the interpretation of a null pronoun is not disrupted by a violation of its preference” to “the interpretation of a null pronoun is not disrupted by a violation of its subject-preference”

**Response 1.18:** We changed this formulation according to the Reviewer’s suggestion.

- p. 34, l. 777-779, change “Polish speakers interpret sentences under the working assumption that the topic of a sentence is the most-prominent, first-mentioned, subject antecedent” to “Polish speakers interpret sentences under the working assumption that the most-prominent referent is the first-mentioned, subject and topic antecedent”

**Response 1.19:** This fragment has been changed according to the Reviewer’s suggestion.

- I did not find Appendix A

**Response 1.20:** We have previously added the Appendix to the submission as a separate file - we were not aware that the Reviewers will not be able to access them. It has been fixed and the Appendix is not added at the end of the manuscript.

**Reviewer #2**

I think that this paper has improved substantially since the previous version, I appreciate the efforts to which the authors have gone to address all comments in the last round. My remaining comments are quite minor. The area that now requires a bit of refinement is the discussion, as you will see from my final few comments.

Page 4 line 69: this example needs a number and a translation

**Response 2.1:** The number has been added to the example. Translation is provided in the gloss in the line following the example sentence.

Page 4 line 74: I wouldn’t describe this as a mismatch between a pronoun and a verb. It is a bit confusing to describe it this way, although I know what you are getting at. Rephrase. I think this issue is made more confusing by the fact that, up to this point in the manuscript, you haven’t indicated exactly what information the pronoun carries in Polish (also this is not clear from the example gloss). This should be clarified.

**Response 2.2:** Thank you for pointing out this issue. The problematic formulation has been replaced with the following one:

*“A consequence of subject-verb agreement is that whenever a pronoun of the verb phrase refers to a less-preferred, non-topical or unexpected antecedent, a speaker needs to adjust the interpretation of the sentence and re-identify the referent of the anaphorical expression.”*

We hope that in this corrected, more descriptive form this fragment is not confusing anymore.

Also, we  have updated the gloss of the example sentence (1) so that it explains what information is carried by the pronoun.

Page 10, lines 217: note that the SMASH algorithm is what Kehler and colleagues are arguing **against**

See also page 38 line 880.

**Response 2.3:** Thank you for spotting this, it was indeed our misinterpretation of Kehler and colleagues’ claims. As the discussion of the SMASH algorithm is not central to explaining the ideas proposed by Kehler and Rhode, we decided that the mention of SMASH can be omitted altogether in the text without substantially affecting the argumentation. It was also not discussed in detail in the Discussion, where it was only mentioned as an example of a certain type of pronoun reference theories (the mentioned p. 38 line 880). As such, we believe that both mentions of the SMASH algorithm of pronoun resolution can be dropped from the text without causing substantial changes to the narration.

Page 15, line 343: I think there is some kind of paste/text error here

**Response 2.4:** Thank you for spotting this, it has been fixed.

Page 15, line 352-354. Without knowing the contents of the Alonso-Ovalle or Chamorro studies, it is very unclear why sentences with null pronouns should be overall more natural than those containing overt pronouns. Explain.

**Response 2.5:** We clarified what the previous studies found and how those findings affect our predictions. In the current version of the text the relevant fragment is the following:

*“Moreover, following the studies of Alonso-Ovalle and colleagues (28) and Chamorro and colleagues (21) who found null pronoun sentences are interpreted as more natural than overt pronoun sentences. Following their results, we expected  to observe a similar pattern of results in our data.”*

Page 16, examples: number the examples, and give a translation as well (it is normal to present both a gloss and a translation)

**Response 2.6:** Numbers and translations have been added to the text.

Page 19, statistical reporting: check if the term “b coefficient” is the correct one for this type of statistical analysis. I think what you normally get from a Bayesian ordinal model is an effect size and a credible interval. Check that you are reporting this correctly.

**Response 2.7**: We would like to thank the Reviewer #2 for this comment. We have reviewed once again the way of reporting the results of the bayesian ordinal models.

The “b coefficient” that we reported in the previous version of the text corresponds to the “Estimate” column from the brms model summary which represents the posterior means (or medians, in the case of the post-hoc tests) of the parameters of the reported bayesian regression mode (“Ordinal Regression Models in Psychology: A Tutorial” by Bürkner and Vuorre, 2018). The β coefficients are usually reported in experiments which use bayesian ordinal models, e.g.:

- Zimmerman et al. (2021): <https://www.frontiersin.org/articles/10.3389/fpsyg.2021.675633/full#h4>
- Skovgaard-Olsen et al. (2019): <https://www.sciencedirect.com/science/article/abs/pii/S0010028518301749>
- Robin et al (2021): <https://doi.org/10.1080/09658211.2021.1895221>

However, the convention is usually to refer to the reported estimates using the “β” notation instead of “b coefficient” so we have adjusted the text by replacing the “b coefficient” with “β”. We have also replaced the reference to credible intervals “CI” with “CrI”, which is less ambiguous (as in frequentist statistics “CI” refers to the confidence intervals).

Page 19, line 446-447: “subject-matching sentences were rated as more natural than object-matching sentences for sentences containing overt pronouns. But in Table 1, the mean rating for subjects is much lower than for objects.

**Response 2.8:** Thank you for spotting this inconsistency. We have corrected the description of the results which is now the following:

*“Direct comparisons between the two Antecedent conditions (i.e., subject- and object-matching sentences) revealed significant effects for sentences containing a null pronoun (b coefficient = -0.42, 95% HPD [-0.62 -0.20]): subject-matching sentences were rated as more natural than object-matching sentences. Significant effects were also found for sentences containing overt pronouns (b coefficient = 0.56, 95% HPD [0.29 0.81]); in the case of which, subject-matching sentences were rated as less natural than object-matching sentences. “*

Page 20, study 1 summary: add some nuance to this paragraph. It sounds here as though you are describing categorical patterns (null pronoun only interpreted as subject and overt only as object), which is not the case.

**Response 2.9:** Thank you for bringing this issue to our attention. We rephrased the summarizing sentence to stress that it is not a categorical pattern but rather a preference. In the current form it is as follows:

*“The results of Study 1 showed that Polish native speakers prefer to interpret the null pronoun of a subordinate clause as referring to the subject of the main clause and the overt pronoun of a subordinate clause as referring to the object of the main clause.”*

Page 25: provide a translation for the example materials

**Response 2.10:** Similarly as in the case of previous example sentences, numbers and translations have been added to the text.

Page 32, line 724: “sentences referring to object antecedent were rated on average as more natural than sentences referring to the subject antecedent”. You can ignore this main effect of antecedent here, as it interacts with pronoun - so the main effect of antecedent is driven entirely by the difference in the object pronouns. (In general, if main effects are qualified by a significant interaction they should not be interpreted on their own)

**Response 2.11:** Thank you for this comment. We agree that the main effect itself is not informative in this case so we have removed the sentence referring to the main effect of Antecedent from the text. Instead, we only talk about the effect of Antecedent in the context of its interaction with the effect of Pronoun so the current version of the description of the results of the naturalness rating in Study 2 is the following:

*“We found a strong effect of Pronoun (β = -0.40; 95% CrI [-0.69 -0.10]): sentences containing null pronouns were rated as more natural than sentences containing overt pronouns. We also found evidence for the interaction of Pronoun and Antecedent effects (β = -1.12, 95% CrI [-1.39 -0.84]). Direct comparisons between the two Antecedent conditions (i.e. subject- and object-matching sentences) revealed that there was no evidence for differences in naturalness score in sentences containing a null pronoun (β = 0.15, 95% HPD [-0.10 0.42]) but there was a strong effect in sentences containing overt pronouns (β = 1.27, 95% HPD [0.99 1.52]): subject-matching sentences were rated as less natural than object-matching sentences.”*

Discussion section: I suggest condensing this, it is quite long and in some places repeats the same point in several ways.

**Response 2.12:** We have shortened the discussion by removing a couple of fragments which repeated the same information and presented similar points. We have also removed the fragment related to the discussion of the spillover effect and lexical access, as suggested by the Reviewer in the following comment. We hope that it is now more condensed and to the point.

Pages 39-40: spillover effect and lexical access. It is difficult to see how this relates directly to the current study. The studies you cite are talking about why effects show up at the spillover region at all (as opposed to a region containing a particular stimulus). But the question you need to answer is why your spillover effect goes in a particular direction. I think this is an entirely different question, so I suggest cutting out this short part of the discussion and references 49-51.

**Response 2.13:** Thank you for this comment. Following your suggestion we have deleted the fragment discussing the spillover effect in the context of lexical access and the corresponding references.

Page 40: you should not make it sound as though Patterson & Felser were the first to propose that pronoun processing occurs in stages over time, this idea has been around in the literature for a very long time

**Response 2.14:** Thank you for bringing our attention to this issue. We have reworded the sentence in which we previously referred to the work of Patterson & Felser to make it clear that we would like to refer to a broader idea. Instead of referring to Patterson & Felser we added a reference to Kehler (2007) who summarizes and critically analyzes the idea of incremental pronoun resolution:

*“This explanation is in line with the idea that pronoun resolution is a process that unfolds over time (for a discussion see: Kehler, 2007): in its early stages, the initial bonding or retrieval are reflected by early fixation measures, but late processes related to the integration with discourse only affect later processing stages.”*

Pages 40-41: I think you have misunderstood the anti-focus effect: it does not represent a cost associated with a “less prominent, non-focused antecedent”

**Response 2.15**: Thank you for spotting this mistake. We decided to remove the mention of the anti-focus effect as the section that mentioned it is supposed to refer, in a broader sense, to experiments that explored the focus effects in pronoun resolution. The corrected version of the paragraph is the following:

*“Previous studies on focus effect in anaphora resolution reported a similar effect: an increased processing cost associated with a topic shift towards a less-prominent antecedent which has been cued by the information structure of the current discourse (10,11,14,15). In this context, our results can be interpreted as showing that Polish speakers automatically interpret the most-prominent antecedent (the first available antecedent or the subject antecedent) to be the topic of a sentence; however, as new information cues another antecedent which is coherent with the current discourse, the reference is shifted.”*
