# Supplementary material for: Pronominal anaphora resolution in Polish: Investigating online sentence interpretation using eye-tracking
Source: PLoS One. 2022 Jan 11;17(1):e0262459. doi: 10.1371/journal.pone.0262459 (PMC8752000; doi:10.1371/journal.pone.0262459)
Supplement: Attachment — Submitted filename: Response to reviewers.docx [file pone.0262459.s005.docx]

We would like to thank the Editor for her comments and suggestions. We have addressed them all and we have had the manuscript proofread by a native English speaker to make sure that there are no grammatical errors left. We hope that the revisions in the manuscript and our accompanying responses successfully address the concerns of the Editor and that after revision you will find our work suitable for publication in PLOS One.

Yours sincerely,

Agata Wolna, Joanna Durlik, Zofia Wodniecka

**Response to the Editor’s comments:**

**Journal Requirements:**

Please review your reference list to ensure that it is complete and correct. If you have cited papers that have been retracted, please include the rationale for doing so in the manuscript text, or remove these references and replace them with relevant current references. Any changes to the reference list should be mentioned in the rebuttal letter that accompanies your revised manuscript. If you need to cite a retracted article, indicate the article’s retracted status in the References list and also include a citation and full reference for the retraction notice.

**Response:** The reference list had not changed since the last round of reviews and we previously checked that it was complete and that the status of the cited work was correct.

**General points**

1. The manuscript still contains several grammatical errors and should be carefully proofread before final submission. Examples found on the first few pages include:

Abstract: "sensitivity to violation" > "sensitivity to a violation"

80: "the reference" > "reference"

**Response E.1.** The specific examples you gave have been changed according to your suggestions. Moreover, the entire text has been proofread by a professional proofreader who is a native English speaker to make sure all other errors were corrected.

2. In-text references should be in angular rather than round brackets, following the usual convention and to avoid literature references being confused with references to linguistic examples.

**Response E.2.** We have updated the literature references in the manuscript and they are now all between angular brackets.

**Specific points**

Abstract: I'm sorry for being picky, but the phrase "ambiguous sentence interpretation task" is still ambiguous as I pointed out before, in that the adjective "ambiguous" can be understood as modifying either "sentence" or "task". Why not simply omit this adjective?

**Response E.3.** Thank you for pointing out this inconsistency. We would rather keep the adjective as it refers to one of the most crucial differences between stimuli used in experiments 1 and 2; however, we have reformulated the problematic sentence and we hope it is clear now. In the corrected version, this fragment is now as follows:

**“***First, we explored preferences in interpretation of null and overt pronouns in ambiguous sentences*.**”**

68: Examples are only numbered from here, and the first two examples on p. 3 are not numbered. Please use example numbering consistently.

**Response E.4.** Thank you for spotting this inconsistency. Numbers have now been added for the first two examples, and the numeration of subsequent examples throughout the text has been updated accordingly.

158: The EPP is not direction-specific and thus does not require the subject to be preverbal. Remove "preverbal" or refer explicitly to Polish, as appropriate.

**Response E.5.** Thank you for pointing this out. The sentence has been changed according to your suggestion and it now reads as follows:

**“***The precise type of the expression in a Spec IP position is further defined by the Extended Projection Principle (EPP), according to which the Spec IP of a given clause must be occupied by a subject [20], which in the case of Polish will always be a preverbal subject***”** (p. 8 lines 161-163).

307 (and elsewhere): Your use of the term "study" is confusing. You report two experiments here which both form part of the same study. Please replace "study" by "experiment" thoughout the manuscript when referring to your experiments.

**Response E.6.** The terminology used in the manuscript has been updated following your suggestion: whenever we refer to one of our experiments, we now use the term “experiment”. The term “study” is used only in reference to previous studies (conducted by someone else).

350: More information about the participants is needed - minimally, their age should be provided.

**Response E.9.** We added the information on participants’ age to the manuscript (p. 15 line 358). In this study we only collected the information on participants’ gender, age and native language, so we do not have any more precise information describing the group in this case.

380: “Qualtrics”? Please provide the URL to this platform or other reference.

**Response E.8.** We now provide the URL to the platform in the manuscript: *“The experiment was conducted via an online survey platform (Qualtrics: www.qualtrics.com).”*

p.24: Consider using a symbol other than an asterisk to indicate dispreference as asterisks normally indicate ungrammaticality.

**Response E.9.** Thank you for bringing this to our attention. We replaced asterisks with “?” to mark dispreference in sentence interpretation in the text.

533: Again, more participant bio-information is needed.

**Response E.10.** In the case of Experiment 2, more information on the participants’ group was provided but only after explaining the exclusion criteria. However, we agree that it was not the best way of providing information. We moved the fragment with the information on the final sample size along with the demographic information to the beginning of the paragraph (p. 23, lines 545-548), and we only discuss the exclusion criteria after giving this information. In the case of Experiment 2, in addition to the information on gender and age, we also provide information on participants’ language background in Table 2 (p. 24 line 569). We only collected precise language background information on participants of Experiment 2 and not Experiment 1 because in the case of Experiment 2 we wanted to be sure that our participants would not be functional and very proficient bilinguals. The reason for restricting the participants’ sample to functional monolinguals was driven by previous findings which show that language experience (immersion in L2 or high proficiency levels) can affect online sentence interpretation and pronoun resolution measured with eye-tracking.

571f.: If this is a footnote or endnote, please format and position it as such.

**Response E.11.** As PLOS one does not allow us to add footnotes to the manuscript, we have moved this information to the main text (p. 24 lines 584-586), directly preceding the example of a sentence’s structure. The fragment explaining the structure of experimental sentences is now as follows:

“*The length of the main clause, as well as the frequency of nouns corresponding to the subject and object antecedents, were also matched between sentences. Note that if a sentence included a null pronoun (which is equivalent to omitting the pronoun), no empty space or other replacement were provided in place of the pronoun in the sentence. Each sentence followed the same structure:*

*subject / verb1 / object / time conjunction (“when”) / pronoun (null* or overt) / verb2 / rest1 / rest2***”**

p.25: How long did the experiment take?

**Response E.12.** The experiment took approximately 45 minutes. We have included this information in the text (p. 26, lines 633-634).

775 (and elsewhere): Why does the word "Studies" start with a capital letter?

**Response E.13.** First, following one of your previous comments, we replaced all occurrences of “study” with “experiment”. Second, when talking about experiments, we use capital letters in some cases to specifically refer to our experiments (i.e., “Experiment 1” / “Experiment 2”). As such, these expressions are used as if they were proper nouns, in contrast to “experiment(s)”, which is used as a common noun. Therefore, we would rather not change the words starting with capital letters to lowercase so as to keep this distinction in the text. However, if you have a strong preference for this change, we will be happy to adopt it.

833ff. "In the second study of the current paper we explored to what extent the pronoun resolution preferences can be modulated by the grammatical constraints of a given sentence. Our results indicate that sentences containing a null pronoun equally easily accommodate verb phrases referring to subject and object antecedents." - It is not clear what is meant by "grammatical constraints" here (a term which replaces the previously used but misleading term "context"). Do you mean a sentence's grammatical properties, its structure, or something else? Secondly, how can verb phrases possibly refer to subject or object antecedents? Please rephrase.

**Response E.14.** We have rephrased this fragment to make it more specific. We have added a sentence to explain what we mean by “grammatical constraints'' and we have replaced the term “verb phrase” with “verb”. After corrections, this fragment reads as follows:

**“***In Experiment 2, we explored the extent to which pronoun resolution preferences can be modulated by the grammatical constraints of a given sentence. In the case of Polish, grammatical constraints refer to grammatical number, person or gender agreement between the verb and its antecedent. Our results indicate that sentences containing a null pronoun accommodate verbs referring to subject and object antecedents equally easily.****”*** (p. 36, lines 858-863)

Fig.1: Why does "Noun Phrase" start with capitals?

**Response E.15.** There was no real reason for that. We have corrected the text in the figure and it no longer starts with capital letters.
