# Supplementary material for: Pronominal anaphora resolution in Polish: Investigating online sentence interpretation using eye-tracking
Source: PLoS One. 2022 Jan 11;17(1):e0262459. doi: 10.1371/journal.pone.0262459 (PMC8752000; doi:10.1371/journal.pone.0262459)
Supplement: Attachment — Submitted filename: Response to reviewers.docx [file pone.0262459.s006.docx]

We would like to thank the Editor for her comments and suggestions. We have carefully read through the manuscript and fixed a number of grammatical and linguistic inaccuracies and mistakes. We have also had the manuscript proofread by a native English speaker to make sure that there are no grammatical errors left. We hope that the revisions in the manuscript successfully address the concerns of the Editor and that after revision you will find our work suitable for publication in PLOS One.

Yours sincerely,

Agata Wolna, Joanna Durlik and Zofia Wodniecka

**Response to the Editor’s comments:**

You have addressed most of my previous comments well enough, but please check your manuscript again carefully for grammatical and linguistic accuracy. Reading through your current manuscript I spotted the following two points, but there may be more.

Thank you for pointing out to this problem. We have carefully read through the manuscript and fixed a number of grammatical and linguistic inaccuracies. We have also asked a professional proofreader (a native English speaker) to read the manuscript again and we have introduced the corrections he has suggested.

214: on one hand > on the one hand

This mistake has been corrected following the Editor’s suggestion.

840ff.: “In Experiment 2, we explored the extent to which pronoun resolution preferences can be modulated by the grammatical constraints of a given sentence. In the case of Polish, grammatical constraints refer to grammatical number, person or gender agreement between the verb and its antecedent. Our results indicate that sentences containing a null pronoun accommodate verbs referring to subject and object antecedents equally easily.” - The reworded passage makes no sense. Verbs cannot refer to subject or object antecedents any more than verb phrases can! It is pronouns that require antecedents. The constraints you mention are not constraints of sentences - I am guessing that what you mean are constraints on referential dependency formation. Please reword as appropriate.

We reformulated this fragment following the Editor’s suggestion. It is now as follows:

*“In Experiment 2, we explored the extent to which pronoun resolution preferences can be modulated by the grammatical constraints on the formation of a referential dependency. In the case of Polish, these constraints refer to the grammatical number, person, or gender agreement between the pronoun and its antecedent. Our results indicate that null pronouns can equally easily be interpreted as referring to subject and object antecedents.” (p.36, lines 843 - 847)*
